# Visual Computation of Material Microstructure and Deformation

**DOI:** 10.3390/ma17122854

**Published:** 2024-06-11

**Authors:** Rongshan Qin

**Affiliations:** School of Engineering & Innovation, The Open University, Walton Hall, Milton Keynes MK7 6AA, UK; rongshan.qin@open.ac.uk

**Keywords:** microstructure, computational method, deformation, microstructure–property relationship

## Abstract

The experimentally obtained material microstructure can be used to calculate a material’s properties and identify microstructure–property relationships. The key procedure to enable this is to interpret the observed microstructure accurately. This work reports on a newly developed computational method to serve such a purpose. The method is based on cubic spline interpolation and a simple search algorithm. Parameterisation was accomplished via the comparison between its preliminary statistical results and the information in a phase diagram. The method was applied to analyse the quenched microstructure of multicomponent and multiphase metallic-oxide materials. The importance of adequate parameterisation is demonstrated. The results provide a good explanation for the experimentally measured electric conductance behaviour. Further application of the method to the deformation of materials is discussed. The algorithms are directly available for the analysis of the three-dimensional microstructure of materials.

## 1. Introduction

Material microstructural characterisation using various microscopes, surface profilers, and wave diffractions results in the production of thousands of images every year. Processing these images can reveal the geometric characteristics of materials at the multiscale, which are closely linked to their properties and processing conditions. Using systematic varied processing parameters, the corresponding changes in the observed microstructure inform of the processing–microstructure relationships. An example can be seen in our recent paper about the effect of various electric processing parameters (current density, frequency, and loading duration) on the phase distribution and grain morphological evolution in cast mould flux [1], where quantitative microstructure interpretation plays an important role in characterising the processing-microstructure relationship. On the other hand, different microstructures give rise to different material properties. Although experimental measurement is the ultimate route to determine the microstructure–property relationship, numerical calculation of a material’s properties from the observed microstructure can provide in-depth knowledge about microstructure–property relationships. Examples include the prediction of the fracture behaviours in brittle materials from their known configuration of phases and compositions using numerical calculation [2,3]. Precise interpretation of the observed microstructural pattern is again the key procedure to identify microstructure–property relationships.

In experimentally obtained microscopic images, such as those obtained from scanning electron microscopes (SEM), the brightness profile reveals morphological information of various phase fields. The contrast in those images is influenced by the interaction between the electron beam and the local surface of the sample. Roughness, chemical composition, and defect density all contribute to the distribution of contrast. Although sample preparation, such as the appropriate polishing and etching, can generate different roughness for different phases and hence improve the contrast, it is often observed that the contrast changes smoothly rather than sharply from one phase to another in these images. This imposes a challenge to the accurate interpretation of the microstructure, e.g., the critical greyscale value to separate two phases. The aim of the current work is to provide a computational and parameterising method to tackle the problem.

The microstructural images obtained from numerical simulations, however, are different from those obtained experimentally regarding the problem described earlier. In numerical calculations, such as those in phase-field simulation [4,5], the physical status of each pixel in the image is fully traced using a phase-field order parameter (*ϕ*), and its value is always known throughout the computation. For example, ϕ<0.1 is frequently used to represent a liquid, ϕ>0.9 is frequently used to represent a solid, and 0.1≤ϕ≤0.9 is frequently used to represent the interface in the simulation of solidification. In experimentally obtained microstructural images, however, the physical status of each pixel is represented by a greyscale value and its meaning needs further identification. The different etching times and accelerating voltage for electrons and facilities can cause the greyscale to alter drastically. Sometimes, comparison of microscopic images at the same location using different characterisation methods can help to clarify the physical status. Examples include the use of both SEM and electron backscatter diffraction (EBSD) images to determine whether two pieces of crystals belong to the same grain according to both their morphological characteristics and their crystallographic orientations. However, this is not always economic or available. This has driven the development of many numerical methods to solve microstructural problems. For example, machine learning using convolutional neural networks has led to some methods to count the number of grains with classified geometric shapes. An example of such a method is the widely used Image J software [6], which was originally developed for biological and medical research to count the number of different types of bacteria and cells in microscopic images. The variation autoencoder is another powerful tool that can convert complex image information into several latent parameters, which can be used to generate artificially similar microstructures to aid material design by varying the values of latent parameters [7]. This can also help quantify the long-standing argument regarding the similarity of microstructural images obtained at different positions in the same sample.

Although it is true that some phases in materials exhibit unique topological morphologies due to atom arrangement and processing conditions (such as undercooling-induced interface instability leading to dendrite formation [8], crystal structure-related interface anisotropy leading to cubic [9] or hexagonal surface formation [10], and displacement transformation-induced plate-shaped martensite [11]), many grains lose these characteristics during subsequent thermomechanical processing, such as deformation [12] and recrystallisation [13]. On the other side, new requirements arise for microstructural representation and interpretation. For example, in heterogeneous materials [14,15], the gradient distribution of grain size, rather than the average grain size, can improve both strength and toughness simultaneously. Conversely, the largest defect, rather than the average size or volume fraction of cracks, inclusions, and pores, is primarily responsible for their detrimental effect on materials [16]. Additionally, the alignment of morphological anisotropic grains, without changes to the size and morphology of any individual grain, can completely alter the materials’ electromagnetic properties [17]. To accurately describe the microstructure of materials, more sophisticated methods beyond conventional statistical analysis are required. Knowledge about the computational phase diagram could be integrated into microstructural calculation to enhance its interpretation. Some microscopic images suffer from high-amplitude noise. This makes it difficult to extract accurate information about the microstructure. Although there are various mathematical algorithms available, such as Bayesian inference [18] and neural networks, they may not always provide fast and reliable solutions. In this work, we implement a cubic spline interpolation and develop a simple search algorithm to fulfil the task. The method helps to denoise images quickly with minimal loss of microstructural information. It is suitable for analysing images obtained from slags, ceramics, and other materials that are too brittle to polish, as well as certain metallic phases, such as the ferritic phase in steels, which are difficult to etch. The developed mechanism for extracting microstructural information in this work aids in predicting the properties of the materials.

## 2. Modelling and Algorithm

The value of a physical quantity (g, e.g., mass density, chemical composition, momentum, etc.) at a position ($r_{i})$ in a continuous space can be calculated by its spatial distribution using the following equation [19,20]:

(1) $$g\left(r_{i}\right)=\int g\left(r\right)W\left(\left|r_{i}-r\right|,\;h\right)dr$$

where $W\left(\left|r_{i}-r\right|,\;h\right)$ is called the kernel or weight function, and h is called the smoothing length. In a limit of h=0, $W\left(\left|r_{i}-r\right|,\;0\right)=\delta \left(\left|r_{i}-r\right|\right)$, Equation (1) becomes the following trivial format:

(2) $$g\left(r_{i}\right)=\int g\left(r\right)\delta \left(\left|r_{i}-r\right|\right)dr$$

where $\delta \left(\left|r_{i}-r\right|\right)=1$ for $r=r_{i}$ and 0 everywhere else. When the space is discretised into elements, Equation (1) becomes the following discrete format [19]:

(3) $$g\left(r_{i}\right)=\sum_{j}\frac{m_{j}}{\rho_{j}}g\left(r_{j}\right)W\left(\left|r_{i}-r_{j}\right|,\;\;h\right)$$

where $m_{j}/\rho_{j}$ is the volume of the element j. In such a mathematical frame, the gradient of a property can be obtained by the gradient of the weight function rather than the gradient of the quantity itself [19].

(4) $$\frac{𝜕g\left(r_{i}\right)}{𝜕x_{\alpha }}=\sum_{j}\frac{m_{j}\left[g\left(r_{j}\right)-g\left(r_{i}\right)\right]}{\rho_{j}}\cdot \frac{𝜕W\left(\left|r_{i}-r_{j}\right|,\;\;\;h\right)}{𝜕x_{\alpha }}$$

where $x_{\alpha }$ is one of the coordinate axes. There are several formats available for the weight function. The most commonly used format is the cubic spline kernel [19,20], which is based on Schoenberg’s piece-wise continuous functions [21]. This kernel gives a weight function for the contribution of a quantity at the radial direction with a distance $\left|r_{i}-r_{j}\right|$ as follows:

(5) $$W(|r_{i}-r_{j}|,\;h)=\left\{\begin{array}{c} \frac{c_{d}}{h^{d}}\left[{\left(2-\frac{\left|r_{i}-r_{j}\right|}{h}\right)}^{3}-4{\left(1-\frac{\left|r_{i}-r_{j}\right|}{h}\right)}^{3}\right]\;\mathrm{for}\;0\leq \frac{\left|r_{i}-r_{j}\right|}{h}\leq 1\\ \frac{c_{d}}{h^{d}}{\left(2-\frac{\left|r_{i}-r_{j}\right|}{h}\right)}^{3}\;\;\;\;\;\;\;\;\;\;\mathrm{for}\;\;1\leq \frac{\left|r_{i}-r_{j}\right|}{h}\leq 2\\ 0\;\;\;\;\;\;\;\;\;\;\;\;\;\;\mathrm{for}\;2<\frac{\left|r_{i}-r_{j}\right|}{h} \end{array}\right.$$

where d is dimension. The coefficient $c_{d}$ takes a value of 1/6, 5π/14, and 1/4π for one-dimensional (1D), two-dimensional (2D) and three-dimensional systems (3D), respectively. Equation (5) introduces a truncation radius that limits the contribution of distant elements to the calculation of the values at a given position. This allows the summation in Equations (3) and (4) to be calculated efficiently using the values at the position and its surrounding neighbours only, thereby reducing computing time.

A microscopic image, regardless of its dimensions, is formed by a bunch of discrete pixels with each pixel being represented by a greyscale value. Different phases have different brightness ranges due to their different chemical constitutions, crystal structures, corrosion behaviours during etching, etc. Those pixels greyscale in the same greyscale range represent a phase. Different phases have their greyscale values falling in different ranges. This type of classification agrees with metallurgical practise. Once the critical greyscale values to separate different phases are defined, one can convert the greyscale distribution to a phase-field order parameter distribution. The interface between different grains can be calculated according to the well-established marching square method. The total interface area and fraction of each phase can be obtained. Using visualisation software such as MatVisual, one can immediately see the phase distribution, grain distribution, and grain morphological characteristics. To know the number of grains and the size of each grain, the following simple grain searching algorithm was developed in the present work. The algorithm starts to search a small unit and then scans the image unit-by-unit to find grains. If a pixel does not belong to any found grains, a new grain is created. As scanning progresses, the previously independent grains that are found to be linked are merged into the same grain. After scanning, the total number of grains in each phase is found, and the number of pixels in each grain represents the volume of the corresponding grain. The spatial configuration of each grain’s pixels provides information about grain morphology, while the length of the outskirt of each grain corresponds to the interface. This procedure provides the required information in the image accurately and efficiently.

## 3. Application, Parameterisation, and Validation

**Figure 1:**
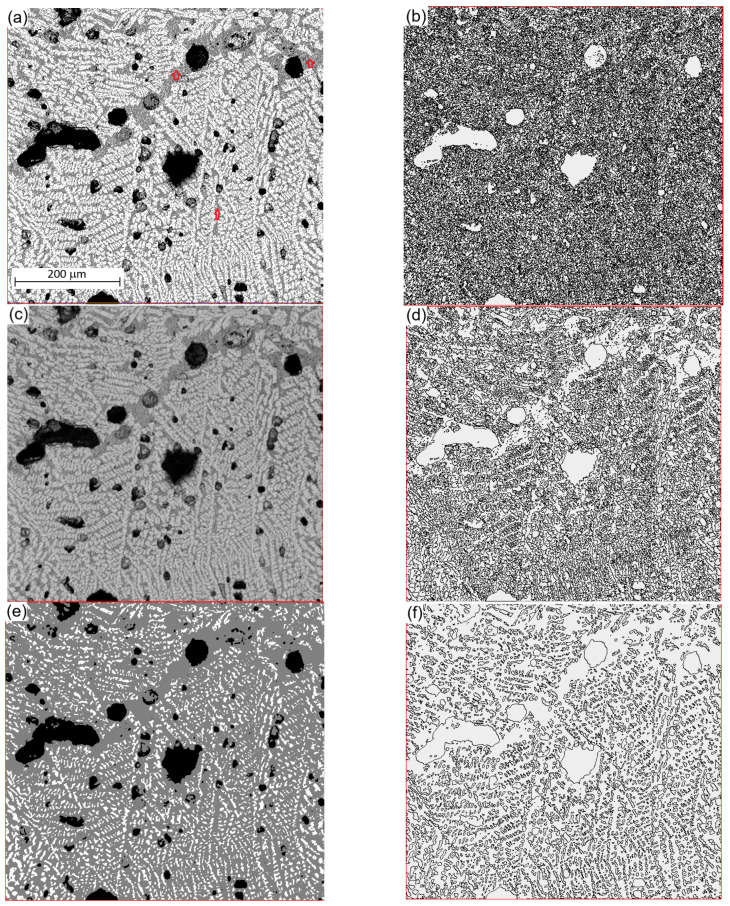
(**a**) Original image and (**b**) its interface; (**c**) smoothed image using h=1 and (**d**) its interface; (**e**) smoothed image using h=2 and (**f**) its interface. The arrows in (**a**) indicate the residual phase.

Figure 1a shows an SEM image (obtained from a Zeiss Supra 55VP FEG SEM manufactured by Carl Zeiss AG) of an as-cast C-27.82CaO-19.87SiO_2_-7.64Na_2_O-7.51Al_2_O_3_-6.56F-5.9MnO-0.96Fe_2_O_3_-0.79MgO-0.31TiO_2_-0.14K_2_O (wt.%) slag [1], which was obtained from the solidification of mould powders. The red lines in the images were added artificially to show the computational frame. This is to avoid including the areas with labels and notations in the SEM images. During sample preparation, the as-received mould powders were heated to 1173 K and maintained for 4 h to remove vapours, carbonaceous and other volatile constituents before being melted in a graphite crucible using an induction furnace. After the induction furnace’s heating was turned off, the molten mould flux was allowed to air cool in ambient conditions. The solidified samples were cut along the longitudinal section, polished, and examined via optical scanning electron microscopy. The image contained three phases: pores (darkest colour), cuspidine (2SiO_2_·3CaO·CaF_2_) primary dendrites (lightest colour), and a mixture of cuspidine, nepheline (NaAlSi_2_O_4_), and other residual elements (colour in between) [22]. Each pixel’s brightness is represented by a greyscale integer ranging from 0 (black) to 255 (white) according to the regulations in computer graphics. A visualisation code package, MetallTools, was developed to pick up greyscale value at each phase, which provides greyscale value for the point that was just mouse-clicked. A random click in a pore, the residual phase, and primary dendrites, separately, gave greyscale values of 11, 123, and 202, respectively. These values can be changed slightly if different locations are clicked. The code package has the functionality to set these values manually. When the critical greyscale value to separate two phases is assumed to be in the middle of two adjacent phases, the pores are in a greyscale range between 0 and 66, those of the residual phase are between 67 and 161, and those of the primary dendrites are between 162 and 255. The interface was calculated using the marching square method and is plotted in Figure 1b.

Figure 1a shows significant noise in each of the three phases. The interface shown in Figure 1b demonstrates the intensity of the noise. For example, the residual phase labelled by arrows in Figure 1a is hardly recognisable in Figure 1b due to the greyscale value fluctuation. Although the phases in Figure 1a are recognisable by the naked eye, it is difficult for a computer to recognise the pattern and perform statistical calculations of the microstructural characteristics without numerical treatment. For this purpose, Equation (3) with h=1 and the kernel format in Equation (5) were implemented to recalculate the greyscale values in each pixel in Figure 1a. The result is shown in Figure 1c and the corresponding interface is shown in Figure 1d. The grain morphology in Figure 1c is much smoother than that in Figure 1c, and all three phases are recognisable in Figure 1d. Figure 1e,f shows the results with h=2. It can be seen that the image is over-smoothed, and the fractions of dendrite and residual phases are clearly changed by the smoothening calculation. More different smoothing lengths with h=3 and 4 were tested. h=1 gives the best result.

**Figure 2:**
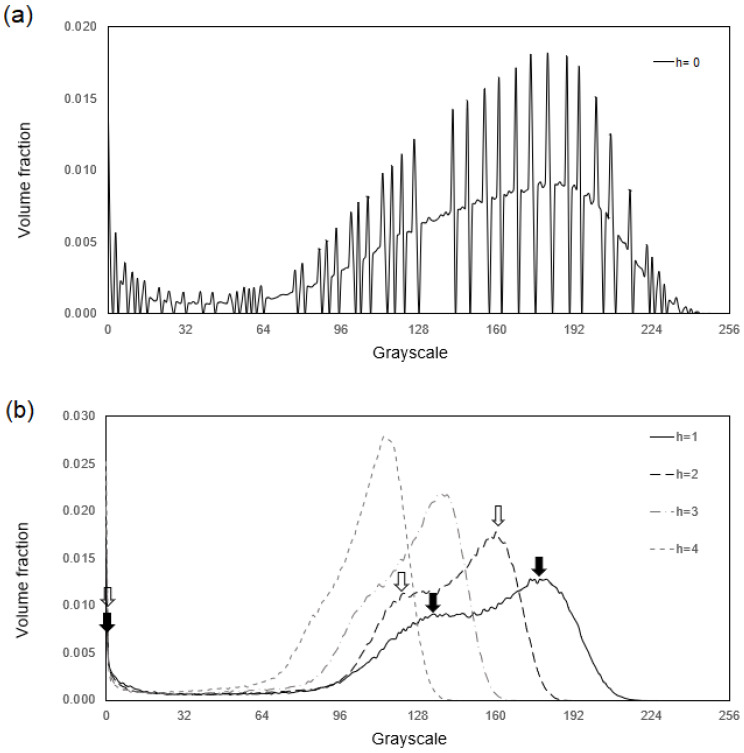
Distribution of pixel greyscale at different smoothing lengths for (**a**) the original image and (**b**) smoothed images. The arrows indicate the peaks.

Figure 2 shows the volume fraction of the pixels and the greyscale value relationship. Figure 2a was obtained from the original image of Figure 1a, and Figure 2b is for those processed images in Figure 1c,e and the other unpresented ones. Instead of the expected three peaks corresponding to the three phases and the area under each peak equalling the corresponding phase’s volume fraction, Figure 2a contains not only high-amplitude noise but only two peaks instead of three. Figure 2b shows that the noise in the smoothed image has been effectively suppressed for all h>0 calculations. In the calculations with h=1 and h=2, some missing peaks caused by noise reappear, as indicated by the arrows. In contrast, excess smoothing lengths with h≥3 smears out some peaks again and moves the peaks toward the average greyscale value in the image, which should be avoided.

**Figure 3:**
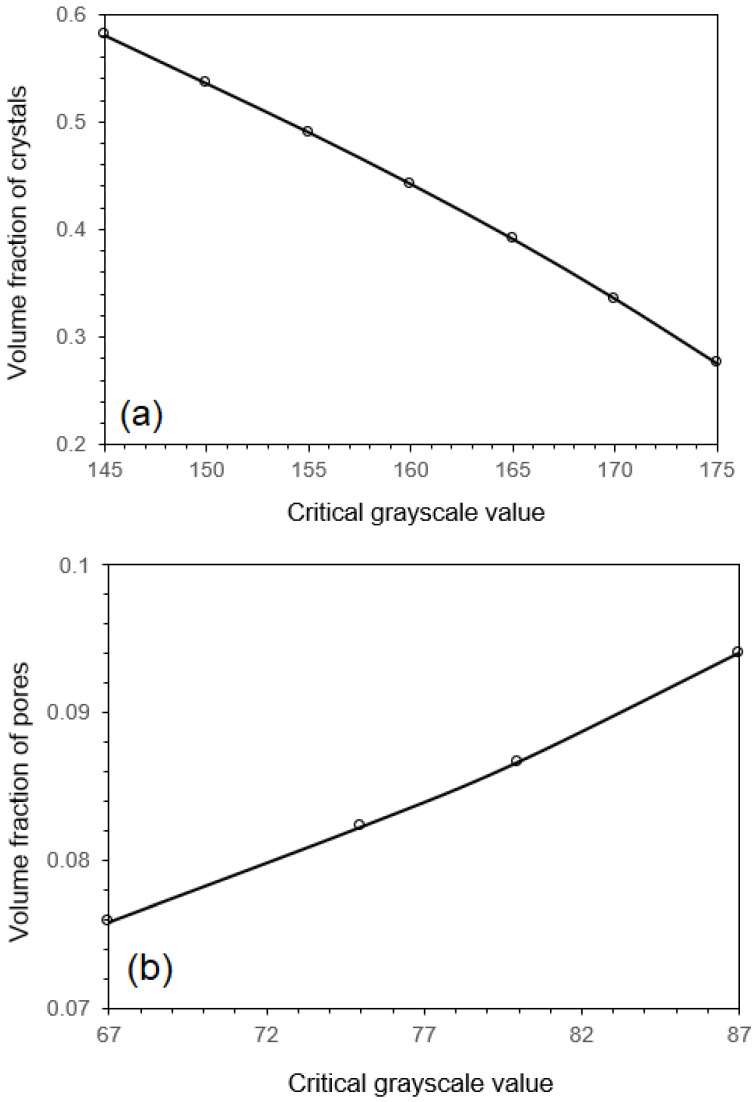
The relationship between (**a**) the volume fraction of primary dendrites and the critical greyscale values, and (**b**) the volume fraction of pores and the corresponding greyscale values obtained from the smoothed SEM image with h=1.

After setting of the smoothing length with h=1, the next group of parameters to be defined is the critical greyscale values. As can be seen in Figure 2b with the curve at h=1, different critical values will give rise to different amounts of phases. To obtain a more precise microstructure description, it is important to set the critical greyscale values correctly so that the amounts of phases represented in the image is correct. However, the peaks in the greyscale distributions shown in Figure 2b are not sharp enough, and the volume fraction of greyscale is not well converged between neighbouring peaks. This raises a need to establish reliable critical greyscale values to obtain precise volume fractions of each phase. To address this, Figure 3 displays the calculated relationship between (a) the volume fraction of primary dendrites and the critical greyscale values and (b) the volume fraction of pores and the corresponding greyscale values in the smoothed image. The figure demonstrates a clear monotonic relationship between the critical greyscale values and the volume fraction of each phase. If the volume fraction of each phase is provided, the critical greyscale values can be calculated straightforwardly using the algorithms proposed in this study and the numerical results presented in Figure 3. To this end, one suggests using the volume fraction of phases provided by computational thermodynamics [23], phase diagram calculations [24], and experimental techniques like X-ray diffraction. By such means, the computational algorithms developed in this study can directly achieve a quantitative assessment of material microstructure.

**Figure 4:**
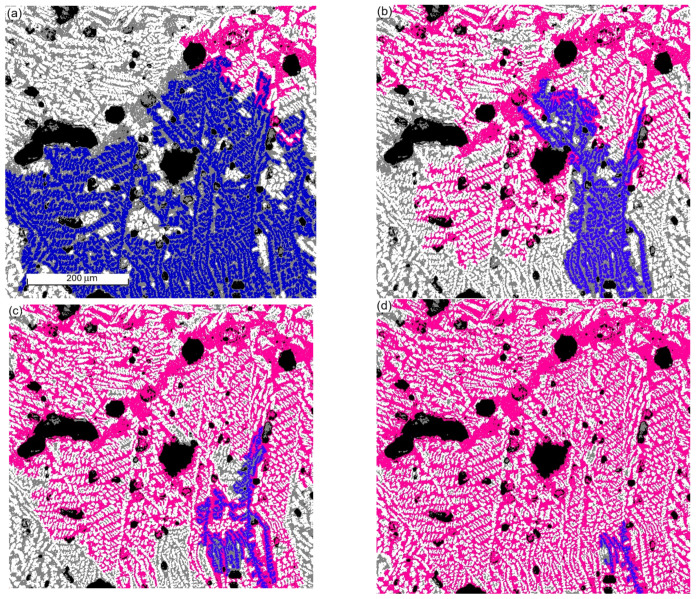
The change in grain distribution at critical greyscale values between the residual phase and primary dendrites at (**a**) 150, (**b**) 155, (**c**) 160, and (**d**) 165. The critical greyscale value between pores and the residual phase is 67. Two grains are highlighted, with one in the residual phase in pink and another dendrite grain in blue.

The importance of the accurate implementation of the volume fraction of phases to determine the critical greyscale values is demonstrated in Figure 4, where the critical greyscale value between pores and the residual phase is set to 67, while that between the residual phase and primary dendrites is set to (a) 150, (b) 155, (c) 160, and (d) 165. Two grains are highlighted: one at the residual phase in pink (around the top-right) and another dendrite grain in blue (around the bottom-right). Both have significantly different sizes from Figure 4a–d. This imposes an important impact on the prediction of the mechanical and physical properties of materials [25]. For example, the crystal phase has better heat conductivity than the other phases [26], where a large-scale crystal skeleton forms a percolation path to conduct heat, while the intersected dendrite by the residual phase is unable to do so.

Figure 4 demonstrates another important fact. When the volume fraction of the dendrite drops slightly, the chance for the grains in the residual phase to link to each other to form a larger interconnected area is increased drastically. The grain in the pink color in Figure 4a occupies only a small corner. However, it spreads to cover a majority area in Figure 4b and becomes dominant in Figure 4c,d. The grain searching algorithm reveals the following information for primary dendrites in Figure 4: (1) the volume fractions are: (a) 0.54, (b) 0.49, (c) 0.44, and (d) 0.39. (2) The largest dendrite grain occupies an area of (a) 113,538 μm^2^, (b) 28,942 μm^2^, (c) 11,511 μm^2^, and (d) 2476 μm^2^. (3) The number of dendrites of significant size is (a) 1, (b) 10, (c) 26, and (d) 173, where a significant size means that the grain area is not in the smallest region if the grain areas between the largest and smallest ones are divided into 10 equal regions. For the residual phase in Figure 4, the maximum domain area is (a) 18,322 μm^2^, (b) 91,090 μm^2^, (c) 143,294 μm^2^, and (d) 189,491 μm^2^, respectively. Although the grain searching algorithm counted the total number of grains and the total grain area in each phase, we avoided mentioning the average grain size in the present case because lots of grains are not fully visible in the image scope. However, it can be used in other images where most of the grains are fully displayed in the view scale. In oxide materials with the chemical constitution discussed in the present work, electrical conduction is carried out by the motion of the cations and anions. The conductivity of the solidified phase is negligible due to the rigid bonds between atoms and molecules. Those residual liquid phase domains entirely entrapped by the solidified phase areas do not contribute to the overall material’s electric conductance. It is well known that electropulsing retards the growth of the phase with high electrical resistance. The reduced amount of dendrite enables the residual liquid phase to form a network and conduct electricity. This partially explains the experimentally observed high conductivity up until a very low temperature.

## 4. Discussion

### 4.1. Computation of Material Deformation Using the Real Microstructure

It is worth pointing out that the present work was merely focused on analysing microstructures rather than investigating material properties. It elucidates the image analysis aspect and its potential applications but does not delve into the direct exploration of material properties. However, accurate interpretation of microstructure can be implemented in the computation of material properties directly. The following is one of the examples of such applications in the computation of material deformation. The applicability of the method to explain the measured material properties was validated by our pulsed solidification. For the original microstructure illustrated in Figure 4a, we observed that the maximum area of the residual phase for the pulsated sample is 35,961 μm^2^ when the fraction of dendrite is around the same. This is much larger than 18,322 μm^2^ reported earlier in this work. The increased area of the largest residual phase explains our experimental measurement. The details of this will be published separately.

There are several models to simulate material behaviours under varying mechanical deformation. The starting point is frequently an artificial microstructure instead of a real one. Examples include the deformation of space-filling polyhedrons and checking for the change in interface area and the length of the edge between two interfacial planes [12,27]. The reason for this is that the interfaces and edges are usually the nucleation sides in the recrystallisation of deformed materials. When the experimentally obtained microstructures are interpreted accurately, the observed true microstructure can be used to calculate material deformation using the following method.

The phase property of each pixel was defined after the visual computational processing in the present work. The coordinates of each pixel can be represented as a vector in space by $U\left(u_{1},u_{2},u_{3}\right).$ Change in the vector after mechanical deformation, $v\left(v_{1},v_{2},v_{3}\right)$, can be calculated by the deformation matrix (S) using the following equation [28]

(6) $$\left(\begin{array}{c} v_{1}\\ v_{2}\\ v_{3} \end{array}\right)=\left(\begin{array}{ccc} S_{11} & S_{12} & S_{13}\\ S_{21} & S_{22} & S_{23}\\ S_{31} & S_{32} & S_{33} \end{array}\right)\left(\begin{array}{c} u_{1}\\ u_{2}\;\\ u_{3} \end{array}\right)$$

In homogeneous plastic deformation, S is a constant matrix throughout the material pixels. The elements of S have different values to represent various types of deformation. Table 1 illustrates the non-zero elements of the S matrix for a few simple deformations [25]. Complicated deformation can be recovered by adequate definition of those elements in the S matrix.

The microstructure evolution in the homogeneous plastic deformation can be obtained via application of Equation (6) to every pixel in the microscopic image. The change om interface area and edge between interfaces can be obtained via application of Equation (6) to the calculated pixels’ positions after the marching square method is used. The procedure is similar to that in our previous paper [12]. The homogeneous plastic deformation is based on strain equivalent assumption. The more generic situation is based on stress equivalent assumption, where the stress balance is calculated according to the constitutional stress–strain relationship of each phase in a material. The accurate classification of phase field in each pixel is more important. The research presented in the present work can be very useful for calculating the residual stress distribution, fracture, and other deformation-induced microstructural evolution in the materials.

### 4.2. Computation of Gradient Using Equation (4)

**Figure 5:**
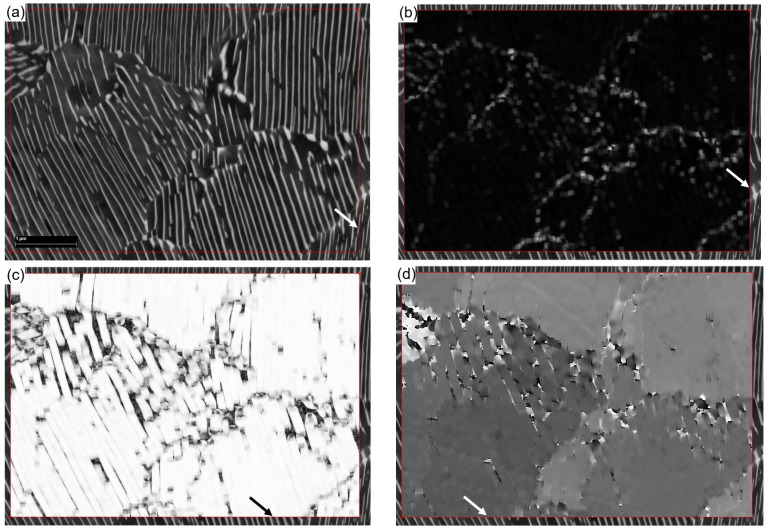
Plotting of (**a**) the pearlitic SEM image, (**b**) λ_2_, (**c**) 1 − λ_2_/λ_1_, and (**d**) ϕ. The larger value is brighter.

Without smoothing treatment, the raw noisy data obtained from Figure 1a can be used to calculate the gradient using Equation (4). This is particularly useful in analysing the aligned microstructure. Since Figure 1a has no such alignment feature, the method was tested for a microstructure from Fe–0.8C–0.2Si–0.5Mn (wt.%) pearlitic steel shown in Figure 5a. The gradient of greyscale along the x- and y-directions is represented by $g_{x}$ and $g_{y}$, respectively. There values can be used to calculate the average values of $g_{x}g_{x}$, $g_{y}g_{y}$, and $g_{x}g_{y}$ within a box of dimension L×L, where L is a length scale that can be chosen based on the microstructural characteristics. The average values of the combined gradient will then be used to calculate morphological orientation and anisotropy via the following three parameters: $\lambda_{1}$, $\lambda_{2}$, and ϕ [29],

(7) $$\lambda_{1,2}=\frac{1}{2}\left(\bar{g_{x}g_{x}}+\bar{g_{y}g_{y}}\right)\pm \frac{1}{2}\sqrt{{\left(\bar{g_{x}g_{x}}-\bar{g_{y}g_{y}}\right)}^{2}+4g_{x}g_{y}}\;$$

(8) $$\phi =\arctan\left(\frac{\lambda_{1}-\bar{g_{x}g_{x}}}{\bar{g_{x}g_{y}}}\right)\;$$

Figure 5b–d shows the computational results for h=1 and L=11. The red-line box in Figure 5 (as denoted by arrows) indicates the chosen boundary for the calculation. Outside the box, the calculation has not been performed, but the properties of pixels might be used if necessary. The selected computational frame allows for selective computing and avoids boundary conditions (although the boundary condition can be treated via the normalisation of the weight function using Vignjevic and Campbell’s equation [30]). L was chosen to cover several interlamellar spacings. $\lambda_{1}$ reflects the morphological orientation. $\lambda_{2}$ reflects anomalies and serves as a method to detect anomalies in laminar microstructures. In Figure 5b, the bright areas with strong anomalies correspond to the original austenite interfaces. The orientation of the laminar structure changes at the original austenite boundary due to the crystallographic orientation relationship between the original austenite phase and the new ferrite phase. Therefore, $\lambda_{2}$ is a suitable parameter to highlight the original austenite interface. The parameter $1-\lambda_{2}/\lambda_{1}$ measures the anisotropy, as is plotted in Figure 5c, where the dark colour indicates more isotropic regions and the white colour indicates more anisotropic microstructures. The local orientation ϕ is plotted in Figure 5d, revealing the original austenite grains. Overall, Figure 5 reveals the rich microstructural characteristics of the pearlitic steel sample and reveals some microstructural transformation histories. The characteristics in the plotting of anisotropy and local orientation are in agreement with those presented in Ref. [29] for different sets of images.

### 4.3. Smoothing Method

All of the pixels in the microscopic images are distributed rectangularly and uniformly. For each pixel under a certain smoothing length h, the total number of effective neighbours (N), the number of different values of weight function M, and the value of the weight function at $\left|r_{i}-r_{j}\right|=0$, $W\left(0,\;h\right)$ are listed in Table 2. When h=1, the property at a pixel is computed using the value of the property at its location and its eight effective neighbours. The latter includes four nearest neighbors and four next-nearest neighbours. The weight factors assigned to these nine sites were found to be similar to those used in the D2Q9 lattice Boltzmann model [31]. These weight factors determine the contribution of each site to the final value of the computed pixel. The cubic spline approach allows for efficient truncation using the smoothing length, resulting in fast numerical calculations. It is also worth noting that the smoothing length does not have to be an integer. This provides flexibility in the calculation.

The effect of smoothing length on microstructural analysis, as shown in Figure 1, can affect the computational results for material properties. For example, material recrystallisation during annealing is related to the amount of interface because the nucleation rate in recrystallisation is higher at the interface. The amount of interface in Figure 1b is 203,834 μm, in Figure 1d, it is 127,132 μm, and in Figure 1f, it is 73,625 μm, respectively. The different number of nucleation sites causes different average grain sizes after recrystallisation and hence the different material strengths according to the Hall–Petch equation.

**Figure 6:**
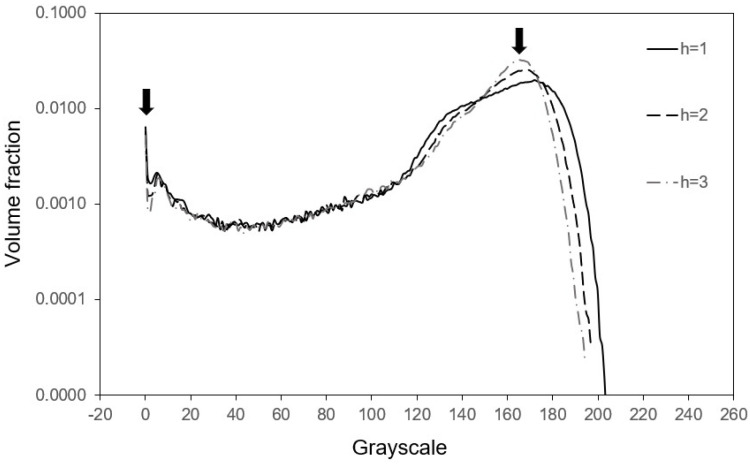
Distribution of pixel greyscale at different smoothing lengths using box average. The arrows indicate the location of peaks. The vertical axis is on a logarithmic scale.

To compare the effectiveness of the cubic spline algorithm with the average smoothing approach, the box smoothing method with $W\left(\left|r_{i}-r_{j}\right|,\;h\right)=1/N$ was tested. The results are presented in Figure 6, which shows that although the noise can be suppressed by the box average, some peaks were smeared out even with h=1. Comparing this with that in Figure 2b, it is obvious that the cubic spline calculation is a better approach than the box average for preserving the peak locations and avoiding excessive smoothing.

**Figure 7:**
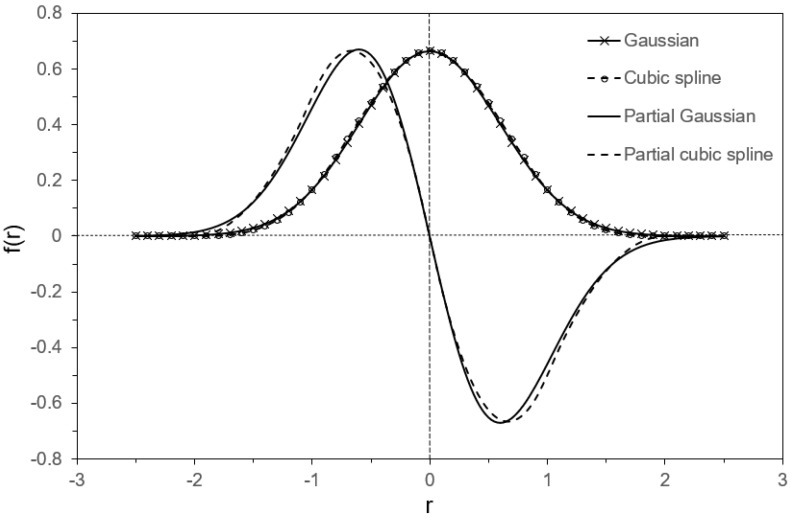
Numerical plotting of Gaussian function with the standard deviation σ = 0.6, one-dimensional cubic spline, and their derivations with regard to the coordinate parameter.

To understand the nature of the cubic spline approximation, the weight function $f\left(r\right)=W\left(\left|r_{i}-r_{j}\right|,\;h\right)=W\left(r,\;h\right)$ was plotted in its one-dimensional format at h=1 and compared with the Gaussian distribution $f\left(r\right)=\left(1/\sigma \sqrt{2\pi }\right)\exp\left(-r^{2}/2\sigma^{2}\right)$ at σ=0.6. The results are plotted in Figure 7. It is evident from Figure 7 that both the cubic spline and the Gaussian distribution curves fit each other well, with their gradients being very close to each other. This similarity between the two distributions suggests that the cubic spline can be used as a smoothing filter similar to the Gaussian blur technique [32], which also filters signals with lower frequencies to suppress noise. It is worth noting that other techniques, such as regularisation in neural networks and Bayesian inference, are also used to remove noise and prevent the overfitting of signals, and often involve the smoothening of high-frequency signals through mathematical processing.

The method presented in this work is also suitable for processing three-dimensional patterns. The cubic spline equation has a three-dimensional format, and the group searching method is unit-based, allowing for direct extension to scan through high-dimensional data.

### 4.4. Scale Factor

It is important to note that the width of an SEM image is often less than 1 mm, and the volume fraction of phases in a material is an average value over a much larger scale. Therefore, applying the volume fraction to an SEM image is only suitable for materials with sufficient homogeneity. In cases where there is insufficient homogeneity at the millimetre scale, multiple SEM images from different positions should be used to determine the critical greyscale value. The computational model presented in this work is also applicable to images obtained through optical microscopy, and applying this method at different scales can improve the accuracy of the results.

## 5. Conclusions

The present work reports a novel computational method to assess material microstructure quantitatively. The method utilises the cubic spline kernel to calculate the smoothed greyscale distribution and its gradient. The method removes noise from the microscope image, recovers missing peaks for phases, and enables the further calculation of material deformation and material properties. A simple group searching algorithm was developed and implemented to identify the grains, their sizes, and spatial configurations. The following conclusions have been reached.

- For the cubic spline kernel $W\left(\left|r_{i}-r_{j}\right|,\;h\right)$, the greyscale distribution in a microscopic image can be calculated using $g\left(r_{i}\right)=\sum_{j}\frac{m_{j}}{\rho_{j}}g\left(r_{j}\right)W\left(\left|r_{i}-r_{j}\right|,\;h\right)$, where the factor $m_{j}/\rho_{j}$ can be determined by the scale bar in the image. The truncation format by means of the smoothing length makes the computation very fast. The calculation not only suppresses the noise in the microscopic image but also recovers the peaks for phases. The method shows better results than that of the box average.
- The gradient of the greyscale can be calculated by $\frac{𝜕g\left(r_{i}\right)}{𝜕x_{\alpha }}=\sum_{j}\frac{m_{j}\left[g\left(r_{j}\right)-g\left(r_{i}\right)\right]}{\rho_{j}}\cdot \frac{𝜕W\left(\left|r_{i}-r_{j}\right|,\;h\right)}{𝜕x_{\alpha }}$. This method allows the greyscale gradient to be calculated by the kernel gradient. The latter is a constant in the regularly arranged pixels, saving significant computing time and allowing the gradient to be obtained from the noisy data directly. The results are implemented to calculate three parameters $\lambda_{1}$, $\lambda_{2}$, and ϕ, which reveals not only the morphological anisotropy and local orientation of the grains but also reveals some microstructural histories.
- The group searching algorithm can accurately identify the different domains in the same phase according to the critical greyscale values; the latter can be determined precisely according to the volume fraction of phases obtained from other means. Group searching can identify the total grain number, the size of each grain, the grain morphology, the ranking of grain, and the location of the grain in microscopic images. The full, detailed microstructural information was identified using the method. This helps to identify the relationships between the microstructure and physical and mechanical properties.
- The cubic spline kernel was found to be similar to the Gaussian formula. This indicates that the underlying denoising mechanism is similar to Gaussian blurring. The principle of removing high-frequency signals shares similarities with artificial neural networks and Bayesian inference. Overall, the developed method presents a promising approach for the quantitative assessment of material microstructure.

## Figures and Tables

**Table 1:** Matrix elements for various deformations [28].

Deformation	Non-zero elements of S
Plain strain deformation	$S_{11}\times S_{33}=1\;$ $S_{22}=1$
Axisymmetric tension	$S_{22}=S_{33}=1/\sqrt{S_{11}}$
Axisymmetric compression	$S_{11}=S_{22}=1/\sqrt{S_{33}}$
Simple shear	$S_{11}=S_{22}=S_{33}=1\;$ $S_{13}\neq 0$

**Table 2:** Cubic spline parameters for various smoothing lengths.

h	N	M	$W\left(0,\;h\right)$
1	8	3	0.4547284
2	44	9	0.1136821
3	108	18	0.0505254
4	192	28	0.0284205

## Data Availability

The software and datasets generated during and/or analysed in the current study are available from the corresponding author upon reasonable request.
